# CULTURAL CONSIDERATIONS AND ATTACHMENT TO PLACE FOR RURAL-DWELLING OLDER ADULTS

**DOI:** 10.1093/geroni/igad104.0432

**Published:** 2023-12-21

**Authors:** Carolyn Adams-Price

**Affiliations:** Mississippi State University, Mississippi State, Mississippi, United States

## Abstract

Mississippi is the only state in the United States where the majority of African Americans live in rural areas. African Americans have lived in rural Mississippi for many generations, despite few resources and health inequities. However, it is a mistake to look at older rural African Americans and see only poverty and illness. Previous research by Rowles and others in Appalachia has indicated that older adults tend to be satisfied with homes in impoverished rural areas. This study examined attachment to home and community in older African Americans living in rural parts of two counties in north Mississippi. Participants were 47 African Americans between 52 and 79 (20 male), with a mean age of 65. Phenomenological analysis for attachment to home yielded 6 themes: home as a peaceful/safe place, legacy/historical roots, attachment to natural features, ownership/I’ve made it my own, and everyday functionality. The first two themes (peaceful/safe and legacy/historical roots) were far more prevalent than the theme of everyday functionality; these findings will be discussed in the context of other researchers’ findings on attachment to place in later life. However, although our participants have experienced discrimination in the larger environment, home is for most of them a place of safety, warmth, and meaning. Attachment to features of the land is also important.

